# Evaluation of MIRU-VNTR for typing of *Mycobacterium bovis* isolated from Sika deer in Northeast China

**DOI:** 10.1186/s12917-015-0402-0

**Published:** 2015-04-11

**Authors:** Li Yang, Chunyu Wang, Haijun Wang, Qingfeng Meng, Quankai Wang

**Affiliations:** College of Chinese Medicinal Materials, Jilin Agricultural University, Xincheng Street No.2888, 130118 Changchun, China; Jilin Entry-Exit Inspection and Quadrant Bureau, Puyang Street No.1301, Changchun, 130062 China; Jilin Sino-ROK Academy of Animal Sciences, Donghua Street No.1699, Changchun, 130600 China

**Keywords:** *Mycobacterium bovis*, MIRU-VNTR typing, Sika deer

## Abstract

**Background:**

Bovine tuberculosis has led to serious economic losses for Sika Deer producers in China. Strategies for controlling the spread of *Mycobacterium bovis* are often hampered by a lack of epidemiological data. Specifically, tracing infections requires the ability to trace back infections, which, in turn, requires the ability to determine isolates with a common source. This study was planned to assess the discriminatory power of each mycobacterial interspersed repetitive unit (MIRU)-variable number tandem repeats (VNTR) locus and evaluate the most appropriate combination of MIRU-VNTR loci for molecular epidemiological studies on Sika Deer in China.

**Results:**

The discriminatory power of MIRU-VNTR typing based on 22 known loci (12 MIRUs, 2 ETRs, 4 QUBs, and 4 Mtubs) were assessed in 96 *Mycobacterium bovis* strains collected sequentially from Sika Deer at a slaughterhouse in northeastern China. We defined four loci (MIRU4, ETRA, QUB11b, and Mtub4) as highly discriminative, eight loci (MIRU2, MIRU23, MIRU27, MIRU31, MIRU39, MIRU40, QUB26, and Mtub21) as moderately discriminative, and three loci (MIRU16, Mtub30, and Mtub34) as poorly discriminative. The final locus showed no polymorphism between strains. MIRU-VNTR typing as a whole was highly discriminative, with an overall allelic diversity of 0.897. Of the loci tested, the four highly discriminative loci and eight moderately discriminative loci proved to be most appropriate for first line typing of *M. bovis* from Sika Deer, with the same resolving ability as all 22 loci (H = 0.897).

**Conclusions:**

MIRU-VNTR typing is quick and effective for typing bovine tuberculosis isolates from Sika Deer in China.

**Electronic supplementary material:**

The online version of this article (doi:10.1186/s12917-015-0402-0) contains supplementary material, which is available to authorized users.

## Background

Sika Deer tuberculosis is caused by the *Mycobacterium tuberculosis* complex (MTBC); most commonly, *M. bovis*. This disease has led to significant economic losses for deer producers in China. However, previous efforts in controlling MTBC have been hampered by a lack of data, as genotyping MTBC members is an important tool in epidemiological analysis for studying the spread of MTBC [1]. In 2001, VNTR genotyping method using MIRU units was developed [2] and used to study the molecular epidemiology of *Mycobacterium tuberculosis* [3]. MIRU-VNTR has high efficiency and reproducibility, because MIRU-VNTR typing results are easier to compare between different laboratories than restriction fragment length polymorphisms (RFLP), which were the previous “gold standard” for MTBC typing [4]. Another advantage of MIRU-VNTR is that it does not require large quantities of template DNA for typing [5].

In addition to *M. tuberculosis*, this technique has also been proven in *M. bovis* in different countries and epidemiologic scenarios [1,6-11]. Earlier studies frequently used the loci designated as mycobacterial interspersed repeat units (MIRU) [2,12] and exact tandem repeats (ETR) [13]. To obtain better resolution, other VTNR loci were investigated [14]. Recently, novel *M. tuberculosis* MIRU-VNTR loci groups, such as the Queen’s University Belfast (QUB) and Mtub groups [15], have been identified. Various combinations of MIRU-VNTR loci have been reported to give better differentiation of *M. tuberculosis* strains [16-19]. However, no specific set of loci have been agreed upon as a standard, and the allelic diversity of loci can vary from country to country and between *M. tuberculosis* complex species, requiring localized selection of suitable loci.

This study was therefore developed to determine the MIRU-VNTR loci most appropriate to use for typing MTBC organisms isolated from Sika deer in China.

## Results

### Analysis of individual MIRU-VNTR locus

MIRU-VNTR typing results of M.*bovis* Isolated in this study was described in Additional file 1. The copy numbers and allelic diversity (*h*) in MIRU-VNTR loci were recorded (Table 1). The diversity analysis showed that the discriminatory power of individual loci differed greatly, with *h* ranging from 0.000 to 0.739. The ETRA, MIRU4, QUB11b, and Mtub4 loci had high discriminatory power (*h* > 0.6). The MIRU2, MIRU23, MIRU27, MIRU31, MIRU39, MIRU40, QUB26, and Mtub21 loci were moderately discriminatory (0.3 ≤ *h* ≤ 0.6). MIRU16, Mtub30, and Mtub34 were poorly discriminatory (*h* < 0.3). Seven loci displayed no allelic diversity.

**Table 1:** **Allelic diversity of individual MIRU-VNTR locus**

**loci**	**No. of copies**										**Allelic diversity**
	**1**	**2**	**3**	**4**	**5**	**6**	**7**	**8**	**9**	**10**	
ETRA	23					50	23				0.608
MIRU2			19		77						0.310
MIRU4	16		32	48							0.607
MIRU23				35	61						0.458
MIRU31							64	32			0.439
MIRU39						32				64	0.439
MIRU40		47			49						0.495
QUB11b	30			22		26	18				0.739
QUB26				37	47	12					0.592
Mtub4			40		7		43	6			0.613
Mtub21	53			23	20						0.590
Mtub30		15			81						0.256
Mtub34		6					90				0.108
MIRU16			81	5	10						0.267
MIRU27		14	55	27							0.567
ETRC							96				0.000
MIRU10								96			0.000
MIRU20	96										0.000
MIRU24				96							0.000
MIRU26					96						0.000
QUB1895					96						0.000
QUB3336			96								0.000

### Evaluation of MIRU-VNTR loci combinations

**Figure 1:**
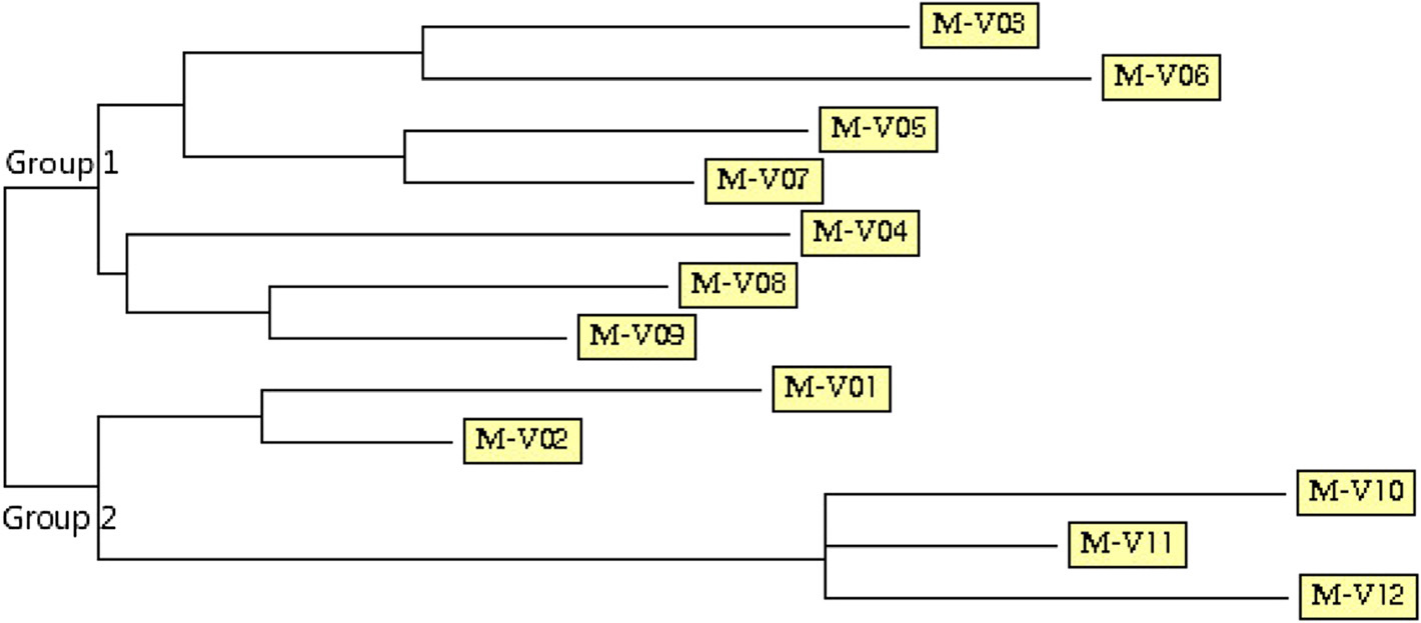
Sample figure title. Group1 are presented with varying MIRU-VNTR types: M-V03, M-V04, M-V05, M-V06, M-V07, M-V08, M-V09.

Based on all 15 MIRU-VNTR loci with non-zero allelic diversity, 12 different types (H = 0.897) were identified; of these, 1 was unique and 11 were clustered. Clusters contained 1 (n = 1) to 17 (n = 1) identical strains (Table 2), and the 96 clinical isolates were classified into two groups (Figure 1). Group 1, which included 7 genotypes, was more complex. This group included isolates from four different regions—ShuangYang, LiaoYuan, TongHua, SongYuan and SiPing—strongly suggesting interregional transmission.

**Table 2:** **Molecular differentiation of isolates by MIRU-VNTR**

**MIRU-VNTR type**	**MIRU-VNTR allelic profile**															**Isolates ID**	**Number of Isolates(N)**
	**ETRA**	**MIRU4**	**QUB11b**	**Mtub4**	**MIRU2**	**MIRU23**	**MIRU31**	**MIRU39**	**MIRU40**	**QUB26**	**Mtub21**	**MIRU27**	**Mtub30**	**Mtub34**	**MIRU16**		
M-V01	1	1	1	7	3	5	7	10	2	5	1	2	2	7	5	ShuangYang1-5	5
M-V02	7	3	1	7	5	5	7	10	2	5	1	2	5	7	3	ShuangYang6-14	9
M-V03	1	3	4	5	5	4	8	10	5	6	1	3	5	7	3	ShuangYang15-21	7
M-V04	6	4	6	7	5	5	7	10	5	4	5	4	5	7	3	ShuangYang22-27; LiaoYuan1-6; TongHua1-5	17
M-V05	1	1	7	7	5	5	7	6	2	5	4	3	5	7	3	TongHua6-13	8
M-V06	6	3	4	3	5	4	8	6	2	4	4	3	5	7	3	TongHua14-28	15
M-V07	6	4	6	3	5	5	7	6	2	5	1	3	5	7	3	TongHua29-32; SiPing1-5	9
M-V08	7	4	1	3	3	4	7	10	5	5	1	3	5	7	3	SiPing6-9; ShuangYang28-36	13
M-V09	1	1	1	3	5	5	7	10	5	5	5	3	5	7	3	ShuangYang37-39	3
M-V10	6	4	7	8	5	5	8	10	5	4	1	4	2	2	4	ShuangYang40-42; SongYuan1, SongYuan2	5
M-V11	6	4	7	7	5	5	8	10	5	6	1	4	2	7	5	SongYuan3-5; ShuangYang43	4
M-V12	7	3	7	8	3	5	8	10	2	6	1	4	2	2	5	SiPing10	1

The discrimination ability was compared with different combinations of loci (Table 3). The four highly discriminatory loci and the eight moderately discriminatory loci, also identified 12 genotypes (H = 0.897). The MIRU loci with moderate discriminatory power (MIRU2, MIRU23, MIRU27, MIRU31, MIRU39, and MIRU40) resolved the 96 isolates into 10 types (H = 0.877). If the MIRU4 and MIRU16 loci were also used, 11 types (H = 0.893) were resolved. With the Mtub4, Mtub21, Mtub30, and Mtub34 loci, 8 types (H = 0.836) were resolved. Based on these results, we consider ETRA, MIRU4, QUB11b, and Mtub4 to be robust enough for initial molecular epidemiological studies when rapid results are of paramount importance.

**Table 3:** **Evaluation of the discriminatory capacity of the combinations of MIRU-VNTR locus**

**Locus combination**	**No. of clusters**	**No. of isolates in individual clusters**	**Hunter–Gaston discriminatory index (HGDI)**
ETRA, MIRU2, MIRU4, MIRU23, MIRU31, MIRU39, MIRU40, QUB11b, QUB26, Mtub4, Mtub21, Mtub30, Mtub34, MIRU16, MIRU27	12	1-17	0.897
ETRA, MIRU4, QUB11b, Mtub4	12	1-17	0.897
MIRU2, MIRU23, MIRU27, MIRU31, MIRU39, MIRU40, QUB26, Mtub21	12	1-17	0.897
MIRU2, MIRU4, MIRU16, MIRU23, MIRU27, MIRU31, MIRU39, MIRU40	11	1-17	0.893
MIRU2, MIRU23, MIRU27, MIRU31, MIRU39, MIRU40	10	1-17	0.877
ETRA, MIRU4, QUB11b, Mtub4	8	6-24	0.836

## Discussion

*M. bovis* is the primary pathogen for tuberculosis in Sika Deer. Because it also infects humans, molecular epidemiologic studies on *M. bovis* are of major importance in tuberculosis prevention. Genotyping of MTBC isolates is a useful tool, not only for regional investigations, such as tracebacks, but to allow comparisons of tuberculosis isolates worldwide [20]. Different typing methods published for *M. bovis* include IS6110, RFLP, VNTR, and spoligotyping [21]. While IS6110 has been the most common, the discriminatory power of IS6110 is generally low for *M. bovis* typing [22,23]. Because the resolution provided by MIRU-VNTR is adequate for most situations and this method is relatively cheap and rapid, it is quickly becoming the standard as the initial step in *M. bovis* epidemiology [14].

The individual discriminatory power of 22 MIRU-VNTR loci were analyzed. The QUB11b locus produced the highest individual discriminatory index (*h* = 0.739), followed by Mtub4, ETRA, and MIRU4. This correlates well with previous studies [14,15].

Mtub loci exhibit good application potential; however, the discriminatory power of Mtub4, Mtub21, Mtub30, and Mtub34 differed greatly, with *h* ranging from 0.108 to 0.613. These loci exhibited a high enough discrimination ability to be useful in early research [24,25], and may prove useful in future *M. bovis* typing studies. The ETRC, MIRU10, MIRU20, MIRU24, MIRU26, QUB1895, and QUB3336 loci showed no allelic diversity in this test. MIRU10, MIRU20, MIRU26 and QUB3336 had similar results in studies on *M. bovis* isolated from Xinjiang region [16]. The published data on *M. tuberculosis* from Ghana [24] and other locations [6,13,14] showed higher allelic diversities than our study, which suggests that not all MIRU-VNTR loci are informative for *M. bovis* strains form Sika Deer in China.

The allelic diversity of MIRU-VNTR loci are higher in our study than previous studies in China [26,27], Published data on *M. tuberculosis* from France [28], South Africa [29], and the United States [30] found that the discriminatory power of MIRU-VNTR loci in Sika Deer is higher than in humans. Although these MIRU-VNTR loci showed enough discrimination ability for genotyping current *M. bovis* isolates, additional studies with new MIRU-VNTRs are needed to ensure accurate genotyping and epidemiological resolution for field studies.

The discriminatory power of MIRU-VNTR loci may vary between countries, geographical regions, and epidemiological scenarios. Analysis of 15 VNTR loci showed the expected results: when compared with previous studies the test had a relatively high Hunter–Gaston discriminatory index (HGDI) and low clustering rates [31-33]. The subset of 15 loci provides increased resolution compared to the original 12 MIRU loci.

The discriminatory power of MIRU-VNTR loci also varies with high allelic diversity. The ETRA, MIRU4, QUB11b, and Mtub4 loci provide the greatest resolution (H = 0.897) for individual loci, while the MIRUs alone (MIRU2, 23, 31, 39, 40, 27, 16, and 4) provided less resolution (H = 0.893). The moderately discriminatory MIRUs (MIRU2, MIRU23, MIRU31, MIRU39, MIRU40, MIRU27) had less resolution alone (H = 0.877) than when combined with the other moderately loci (QUB26 and Mtub21), which had the greatest amount (H = 0.897). Therefore, the discriminatory power of VNTR loci appears greatly improved by applying novel combinations of VNTR loci.

The 15 VNTR loci methodology is a highly discriminatory method for first line typing of *M. bovis* isolated from Sika deer and should be considered as a replacement for the original 12 MIRU loci method. However, for in-depth studies, we recommend using ETRA, MIRU4, QUB11b, and Mtub4 loci as a first set to obtain initial molecular epidemiology data quickly before proceeding on to the full set of loci.

## Conclusions

The results of this study will be used in a molecular epidemiology study to identify risk factors for recent transmission of Sika Deer tuberculosis in China. VNTR studies will also help in tracing infection sources during Sika deer tuberculosis outbreaks and help control transmission between Sika Deer and humans. Finally, this will improve Chinese Sika Deer cultivation by reducing losses due to MTBC infections.

## Methods

### Bacterial strains

A total of 96 *M. bovis* strains were used in this study. These strains were isolated from samples collected at the slaughterhouse for Sika Deer in China covering five different regions: Shuang Yang, Liao Yuan, Tong Hua, Si Ping, and Song Yuan between 2008 and 2012. All 96 strains were characterized by real-time PCR [34].

### Ethical considerations

This research was performed according to the international, national and institutional rules regarding animal experimentation. The research was approved by the Institutional Animal Care and Use Committee of Jilin Agricultural University (reference number:2008627).

### Preparetion of chromosomal DNA

DNA was extracted with the method followed. Cells were suspended in 400 μL TE buffer (10 mM Tris-CI and 1 mM EDTA at pH 8.0) and inactivated at 80°C for 30 min followed by centrifugation at 5000 × g. The supernatant was discarded and the pellet resuspended in 400 μL TE buffer and 45 μL 10% SDS. This was heated in a 37°C water bath for 2 h after incubation with 3 μL of 20 μg/mL proteinase K. The suspension was re-centrifuged and the supernatant extracted using an equal volume of phenol/chloroform/isoamyl alcohol (25:24:1 [v/v]). The resulting supernatant was diluted with a double volume of anhydrous ethanol for 10 min and precipitated at −20°C after centrifugation at 12000 × g for 20 min. The precipitate was washed with 70% ethanol twice, and the ethanol evaporated at room temperature for 20 min. The final pellet was dissolved in 30 μL sterile deionized water and stored at −20°C until use.

### PCR amplification

The primers and designations used in this study for each MIRU-VNTR locus are described by Supply *et al*. [14]. The primer sequences and PCR product sequencing in this study was described in Additional file 2. PCR reactions were performed using the method described in Table 4. The reaction mixture for all loci contained 2 μL *M. bovis* DNA, 0.4 μΜ of each primer pair, 25 μL EmeraldAmp MAX PCR Master Mix (Takara Biomedical Technology, Beijing, China) and 22.6 μL ddH_2_O.

**Table 4:** **PCR conditions for MIRU-VNTR locus**

**Set and multiplex**	**PCR conditions**
ALL MIRUs	Start at a denaturing step of 15 min at 95°C, after denaturation, the PCR was performed for 40 cycles of 1 min at 94°C, 1 min at 59°C and 1.5 min at 72°C. Terminated by an incubation of 10 min at 72°C.
ETRA, C and QUB26	Start at a denaturing step of 12 min at 95°C, after denaturation, the PCR was performed for 40 cycles of 0.5 min at 94°C, 1 min at 60°C and 2 min at 72°C. Terminated by an incubation of 7 min at 72°C.
QUB3336, 1895, 2163	Start at a denaturing step of 15 min at 95°C, after denaturation, the PCR was performed for 40 cycles of 0.5 min at 94°C, 1 min at 55°C and 2 min at 72°C. Terminated by an incubation of 7 min at 72°C.
Mtub4, 21, 30, 34	Start at a denaturing step of 15 min at 95°C, after denaturation, the PCR was performed for 40 cycles of 1 min at 94°C, 1 min at 50°C and 0.5 min at 72°C. Terminated by an incubation of 7 min at 72°C.

### Allelic diversity and VNTR typing

The individual allelic diversity (*h*) of each VNTR locus was calculated using the following equation: *h* = 1-∑*xi*^2^[*n*/(*n* − 1)], where *n* is the number of isolates and *xi* the frequency of the *ith* allele at the locus [35]. We considered *h* > 0.6 as highly discriminative, 0.3 ≤ *h* ≤ 0.6 as moderately discriminative, and *h* < 0.3 as poorly discriminative [36]. The HGDI was calculated as previously described [37]. The HGDI varies between 0.00 and 1.00 and represents the discriminatory power for VNTR loci in combination. Clustering analyses were performed using the online tool at http://www.MIRU-VNTRplus.org.

### Availability of supporting data

Dataset doi:10.6070/H4HX19PG

https://mynotebook.labarchives.com/share/wchunyu1981/MjIuMXw4NDk3MC8xNy01MC9UcmVlTm9kZS8zNjIxMDY0ODMwfDU2LjE=.

## Additional files

Additional file 1:
**MIRU-VNTR type of M.** ***bovis*** **Isolated in this study.** Additional file 1 including the MIRU-VNTR typing results of M.bovis Isolated which was used in this study. Additional file 2:
**PCR primer sequences and PCR product sequencing in this study.** Additional file 2 including PCR primer sequences of MIRU-VNTR loci, and the result of PCR product sequencing in this study.

## Abbreviations

MIRU-VNTR: Mycobacterial Interspersed Repetitive Units - variable number of tandem repeat
PCR: Polymerase chain reaction
HGDI: Hunter–Gaston discriminatory index
